# Looking Beyond Glycaemic Control: Real‐World 12‐Month Cardiometabolic and Retinopathy Outcomes Following Omnipod 5 Initiation in People With Type 1 Diabetes

**DOI:** 10.1111/dom.71129

**Published:** 2026-07-20

**Authors:** Eleanor McNally, Iona Goodman, Rebecca Morris, John Maliekkal, Dalha Saeed, Hao Wei Tan, Simon Berry, Jackie Elliott, Ahmed Iqbal

**Affiliations:** ^1^ School of Medicine and Population Health, University of Sheffield Sheffield UK; ^2^ Sheffield Teaching Hospitals NHS Foundation Trust Sheffield UK

**Keywords:** continuous glucose monitoring (CGM), diabetes complications, diabetic retinopathy, insulin pump therapy, real‐world evidence, type 1 diabetes

## Abstract

**Aims:**

To evaluate the real‐world effectiveness, safety, and tolerability of the Omnipod 5 hybrid closed‐loop system in adults with type 1 diabetes (T1D) with a novel focus on cardiometabolic markers alongside diabetic eye disease and renal function.

**Methods:**

A single‐centre, retrospective analysis of adults with T1D using Omnipod 5 was conducted. Case notes were reviewed for routinely collected clinical data. Continuous glucose monitoring (CGM) metrics were obtained from cloud‐based platforms at 3, 6 and 12 months. Paired data were analysed with two‐tailed *t*‐tests or Wilcoxon signed‐rank, with results presented as mean (SD) or median (IQR).

**Results:**

One hundred individuals were included (63% female). At baseline, median age was 28 (23.0–37.8) years, BMI 27.0 (22.3–30.0) kg/m^2^, diabetes duration 16.0 (9.0–24.8) years and mean HbA1c 68 (±18.0) mmol/mol. Over half (52%) transitioned from multiple daily injections. Significant glycaemic improvements were observed by 3 months. Improvements were durable to 12 months, with mean HbA1c falling from 68.5 to 58.8 mmol/mol (*p* < 0.001, *n* = 75) and time in range (TIR) increasing from 43.4% to 58.3% (*p* < 0.001, *n* = 86). The greatest glycaemic improvements were observed in those with lower TIR at baseline. Retinopathy progression occurred in a minority of 10 individuals, all of whom had a baseline HbA1c > 53 mmol/mol.

**Conclusions:**

Initiation of Omnipod 5 was associated with durable glycaemic improvements. Preliminary data generate the hypothesis that retinopathy progression may be concentrated among individuals with baseline HbA1c above target range. Prospective studies are required to evaluate long‐term retinal and cardiometabolic outcomes while controlling for concomitant weight and lipid‐lowering therapies.

## 1 Introduction

The global burden of type 1 diabetes (T1D) is vast and projected to rise to up to 17.4 million cases by 2040 [1]. Over the past decade, technological advances have transformed the management of T1D. In clinical trials, hybrid closed‐loop (HCL) systems have been highly effective at improving glycaemic outcomes [2]. These systems are now recommended as standard of care for people with T1D (pwT1D) by both the American Diabetes Association and the European Association for the Study of Diabetes [3, 4].

Following the National Institute for Health and Care Excellence (NICE) Technology Appraisal (TA943) in 2023, the Omnipod 5 HCL system was introduced across routine clinical practice in the United Kingdom [5]. Despite clinical trials demonstrating substantial improvements in HbA1c and continuous glucose monitoring (CGM) metrics, real‐world data have revealed challenges in achieving international consensus targets [6, 7]. This discrepancy likely reflects the strict eligibility criteria used in the pivotal trials, which excluded individuals with high baseline HbA1c, frequent severe hypoglycaemia or recent diabetic ketoacidosis (DKA) [2]. Given that these individuals are now explicitly eligible for HCL systems under NICE guidance, there is an urgent need to evaluate clinical effectiveness in these under‐represented populations [5]. Moreover, there is growing recognition that HCL systems should be evaluated in socio‐economically and ethnically diverse populations, to maximise generalisability and identify potential inequities in access and outcomes [8, 9].

Beyond clinical effectiveness, the real‐world safety profile of Omnipod 5 warrants careful evaluation. People living with T1D have a significant risk of developing microvascular and macrovascular complications [10]. Among these, diabetic retinopathy is the leading cause of visual loss worldwide, affecting over 100 million people [11]. Early worsening of diabetic retinopathy (EWDR) has previously been observed in pwT1D following intensive insulin therapy [12]. However, it remains unclear whether contemporary HCL systems confer a significant risk of EWDR. Current evidence is limited to cohorts evaluating alternative HCL systems [13, 14, 15, 16, 17, 18]. Notably, despite the widespread adoption of Omnipod 5, data on retinopathy outcomes have not been published to date. This evidence gap extends beyond retinopathy to the broader cardiometabolic and renal effects of Omnipod 5, underscoring the need for further real‐world data to inform clinical practice [19].

This study aims to evaluate real‐world clinical effectiveness, safety and tolerability outcomes in individuals with T1D following Omnipod 5 initiation. Emphasis is placed on outcomes relating to diabetic eye disease, cardiometabolic parameters and renal function, which have not previously been reported for this HCL system.

## 2 Materials and Methods

### 2.1 Study Design

A single‐centre, retrospective study was conducted to evaluate clinical outcomes in pwT1D using Omnipod 5 at Sheffield Teaching Hospitals NHS Foundation Trust, a large tertiary centre in the North of England. The study was registered as a service evaluation and approved by the Clinical Effectiveness Unit Project Panel as an institutional retrospective case‐note review of electronic health records (Reference 12 262). As the project analysed routinely collected clinical data within a registered service evaluation framework, formal Research Ethics Committee approval was not required under UK governance arrangements.

A retrospective design was chosen to facilitate granular data collection without influencing the true real‐world behaviour of Omnipod 5 users or clinicians. To address potential confounding from concomitant therapies, sensitivity analyses were performed.

The primary outcomes were pre‐defined as changes in HbA1c and CGM‐derived metrics. Secondary outcomes included HCL continuation, time in automated mode, bolus frequency, changes in total daily insulin dose (TDD), weight, body mass index (BMI), blood pressure, lipid profiles, renal function, and severity of diabetic eye disease.

### 2.2 Participant Identification

Participants were identified through the trust's adult pump service. The cohort comprised pwT1D aged > 16 years who initiated Omnipod 5 for the first time between July 2023 and October 2024. To maximise the generalisability of this real‐world cohort, no specific exclusion criteria were applied. A random sample of 100 participants was selected from the 377 eligible individuals in the pump service. To ensure the random sample was representative of the wider clinic population, baseline demographics of the selected cohort (*n* = 100) were compared with an independent sample from the remaining clinic population (*n* = 277).

The sampling strategy reflected the need for detailed manual extraction of granular clinical data, including cardiometabolic parameters and retinopathy gradings, which are under‐reported in the Omnipod 5 literature. The sample size was therefore chosen to balance depth and feasibility of data collection while remaining representative of the clinic population, and is broadly consistent with other real‐world analyses [20, 21].

### 2.3 Data Sources

Retrospective data collection was undertaken between August 2024 and August 2025. Electronic case notes were reviewed for baseline characteristics, anthropometric data and Index of Multiple Deprivation (IMD) scores [22]. Socioeconomic deprivation was quantified using the 2019 English IMD, an established area‐level measure based on residential postcode. The IMD combines seven domains (income, employment, health and disability, education, crime, barriers to housing and services, and living environment) into a composite deprivation score. Discharge summaries were reviewed to identify admissions in the year prior and following HCL initiation. An admission was defined as any emergency department presentation or inpatient stay. Lab results were accessed through the Integrated Clinical Environment (ICE) system used across primary and secondary care. Retinopathy (R0–R3) gradings were obtained from the UK National Diabetic Eye Screening Programme and supplemented by local ophthalmology records for individuals with concurrent specialist follow‐up [23]. Grading was undertaken by independent assessors with arbitration for discrepant findings. Retinopathy gradings were extracted by a blinded data extractor who did not have access to the wider dataset. Retinopathy progression was defined as any increase in retinopathy grade relative to baseline, by at least one level (i.e., R1–R2), at any follow‐up timepoint.

Glycaemic metrics were obtained through CGM cloud‐based platforms, including Glooko, Dexcom Clarity, and LibreView. Validated glycaemic metrics were extracted for the 14‐day period prior to HCL initiation and at 3‐, 6‐ and 12‐month timepoints. Other clinical outcomes were matched to the following time‐points: 3 months (±6 weeks), 6 months (±12 weeks) and 12 months (±12 weeks), or presented with a median follow‐up duration.

### 2.4 Statistical Analysis

Statistical analyses were performed using the Statistical Package for the Social Sciences (IBM, version 29.0.2.0). Data visualisation was undertaken using RStudio (version 2025.09.2+418) and Python (Matplotlib). Baseline data were summarised as mean (SD) or median (IQR) unless otherwise stated. Selection of statistical tests was guided by the distribution of the differences between paired data points. Normality was assessed through visual inspection of histograms alongside skewness and kurtosis values. Two‐tailed paired *t*‐tests were used to analyse normally distributed data. The Wilcoxon signed‐rank test was applied to non‐parametric data. Individuals who discontinued Omnipod 5 during the 12‐month follow‐up period or lacked paired data were excluded from paired longitudinal analyses. Adverse events were analysed across the full cohort (*n* = 100), with admissions expressed as episodes per person per year (PPPY). Where applicable, Pearson's correlation coefficient was used to examine relationships between clinical metrics.

To reduce potential confounding in lipid profile analyses, statin prescriptions were reviewed at baseline and follow‐up. No equivalent review was undertaken for anti‐hypertensive medication, as blood pressure management is primarily undertaken in primary care and medication changes are not routinely documented in diabetes clinic notes.

## 3 Results

### 3.1 Population Demographics

A total of 100 pwT1D who started Omnipod 5 between July 2023 and October 2024 were included in the analysis. The cohort was predominantly of White ethnicity (76%), with a median age of 28.0 (23.0–37.8) years, median diabetes duration of 16 (9.0–24.8) years and mean HbA1c of 68 (±18.0) mmol/mol (Table 1). There were no statistically significant differences in the baseline demographics between the selected cohort (*n* = 100) and the remaining clinic population (*n* = 277). A detailed breakdown of ethnicity data is provided in Table S1, presented in line with the Office for National Statistics 2021 Census results [24].

**TABLE 1:** Baseline characteristics of Omnipod 5 starters.

Characteristic	Sample (*n* = 100)	Remaining clinic population (*n* = 277^e^)	*p*
Age,^a^ years	28 (23.0–37.8)	28.0 (23.5–40.0)	0.444
Female sex, *n* (%)	63 (63)	173 (62.5)	1.000
IMD,^b^ ranks 1–10	5 (2–8)	5.0 (2.0–8.0)	0.886
Quintile 1, *n* (%)	29	29.5	
Quintile 2, *n* (%)	19	11.9	
Quintile 3, *n* (%)	14	21.6	
Quintile 4, *n* (%)	16	18.7	
Quintile 5, *n* (%)	22	18.3	
BMI, kg/m^2^	27.0 (22.3–30.0)	25.7 (22.8–29.6)	0.624
Weight, kg	75.8 (62.9–87.3)	75.9 (66.0–89.5)	0.203
Diabetes duration, years	16.0 (9.0–24.8)	15.2 (8.4–24.9)	0.388
HbA1c, mean (SD), mmol/mol%	68 (±18.0) 8.4 (±1.6)	67.0 (±16.9) 8.3 ± 1.5	0.670
HbA1c, median (IQR), mmol/mol%	64 (55–79) 8.0 (7.2–9.3)	64 (55–79) 8.0 (7.2–9.3)	
Ethnicity, *n* (%)			
White	76 (76)	247 (89)	
Asian or Asian British	4 (4)	10 (3.6)	
Black, Black British, Caribbean or African	4 (4)	5 (1.8)	
Mixed or multiple ethnic groups	2 (2)	1 (0.4)	
Other ethnic group	6 (6)	3 (1.1)	
Unknown	8 (8)	11 (4.0)	
Concomitant long‐acting insulin,^c^ *n* (%)	15 (15)		
Previous insulin delivery,^d^ *n* (%)	MDI: 52 (52)		
	CSII: 41 (41)		
	HCL: 7 (7)		

*Note:* Baseline demographics and clinical characteristics of the selected cohort (*n* = 100) were compared with the remaining clinic population (*n* = 277). Continuous variables were analysed using either Mann–Whitney *U* test (non‐parametric data) or Welch's *t*‐test (normally distributed data; HbA1c). Categorical variables were compared using chi‐square test (gender). *p*‐values: *p < 0.05, **p < 0.01, ***p < 0.001.

Abbreviations: BMI, body mass index; CSII, continuous subcutaneous insulin infusion; HCL, hybrid closed loop; IMD, index of multiple deprivation; MDI, multiple daily injections.

^a^ Age was determined from case notes at Omnipod 5 initiation.

^b^ IMD scores were determined from postcodes, with Decile 1 indicating greater relative socioeconomic deprivation.

^c^ Concomitant long‐acting insulin was prescribed alongside Omnipod 5 at the clinician's discretion.

^d^ Previous insulin therapy refers to mode of insulin delivery immediately prior to pump start.

^e^ Sample sizes varied in the remaining clinic population due to incomplete data in the routine clinic dataset: Gender (*n* = 277), IMD (*n* = 268), weight (*n* = 189), diabetes duration (*n* = 202), BMI (*n* = 156) and HbA1c (*n* = 178).

Over half (52%) of this cohort transitioned from multiple daily injections (MDI), while seven individuals switched from other HCL systems. The majority had previously completed structured diabetes education (55%), with 52% having undertaken the Dose Adjustment for Normal Eating (DAFNE) course [25].

A subset of individuals (*n* = 15) initiated Omnipod 5 alongside concomitant long‐acting insulin. In our practice, concomitant long‐acting insulin is prescribed at the clinician's discretion at approximately 5%–33% of the TDD to mitigate the risk of DKA, particularly in individuals who are pump‐naïve and in those with high total daily insulin requirements. In the latter case, this approach may also help ensure the pod lasts the full 3 days, thus improving cost‐efficiency.

### 3.2 System Characteristics

The most common sensor choice was Dexcom G6 (*n* = 96), with Freestyle Libre 2+ (*n* = 3) and Dexcom G7 (*n* = 1) also utilised. Insulin use comprised Novorapid (*n* = 75), Humalog (*n* = 23) and Fiasp (*n* = 2).

At 12 months, Omnipod 5 users spent 98 (91–100) % of their time in automated mode and on average received 4.1 (2.5–5.3) boluses per day (*n* = 87 paired). Compared to baseline (48.6 ± 21.2 units, *n* = 88), there was no significant change in TDD of insulin at 3, 6 or 12 months.

### 3.3 CGM‐Derived Metrics

**FIGURE 1:**
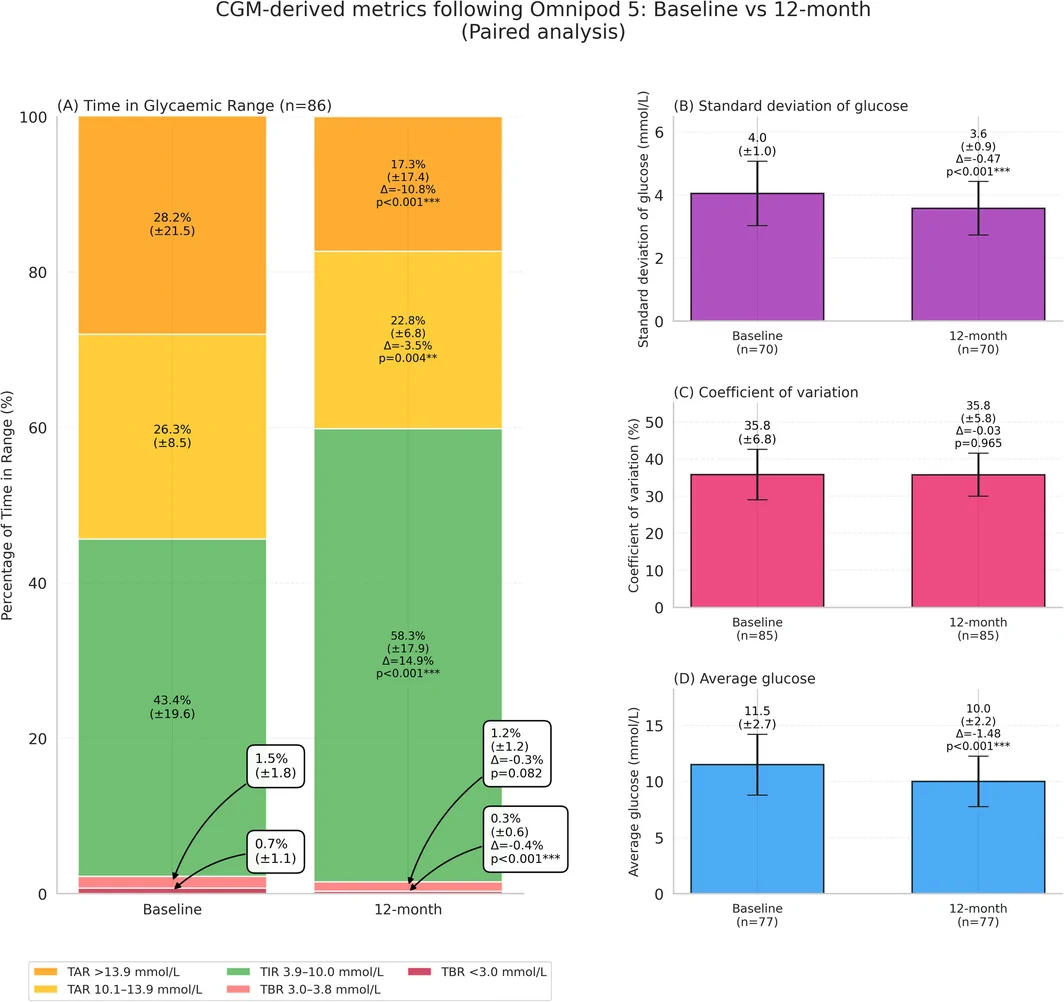
CGM‐derived metrics at baseline and 12 months following Omnipod 5 initiation. Individuals with paired CGM data at baseline and 12‐month follow‐up were included in the analysis. Two‐sided paired *t*‐tests were used to compare baseline and 12‐month metrics. Data presented as mean (SD). Panel A illustrates percentages of time spent in glycaemic ranges: Time above range (TAR) > 13.9 mmol/L (*p* < 0.001***), TAR 10.1–13.9 mmol/L (*p* = 0.004**), time in range (TIR) 3.9–10.0 mmol/L (*p* < 0.001***), time below range (TBR) 3.0–3.8 mmol/L (*p* = 0.082) and TBR < 3.0 mmol/L (*p* < 0.001***). Additional CGM metrics: (B) standard deviation of glucose, (C) coefficient of variation and (D) average sensor glucose. Mean differences (Δ) and 95% confidence intervals (CIs) shown with two‐sided *p* values (*p < 0.05, **p < 0.01, ***p < 0.001).

Paired CGM data were obtained at 3 (*n* = 88), 6 (*n* = 87) and 12 (*n* = 86) months respectively. There was a statistically significant improvement in time in range (TIR; 3.9–10 mmol/L) by 3 months, which was durable across all time points. Full 12‐month glycaemic metrics are illustrated in Figure 1, with 3‐ and 6‐month data provided in Table S1.

To explore factors associated with glycaemic change, correlation analyses were performed. Baseline TIR was moderately correlated with TIR at 12 months (*r* = 0.44, *p* = 0.001). There was a strong inverse correlation between baseline TIR and ΔTIR at 12 months (*r* = −0.59, *p* < 0.001), indicating the most profound improvements in glycaemic control were observed in those with lower baseline TIR. There was no significant association between baseline TIR and IMD decile (*r* = −0.006, *p* = 0.955) or 12‐month ΔTIR and IMD decile (*r* = 0.091, *p* = 0.406).

### 3.4 HbA1c Outcomes

**FIGURE 2:**
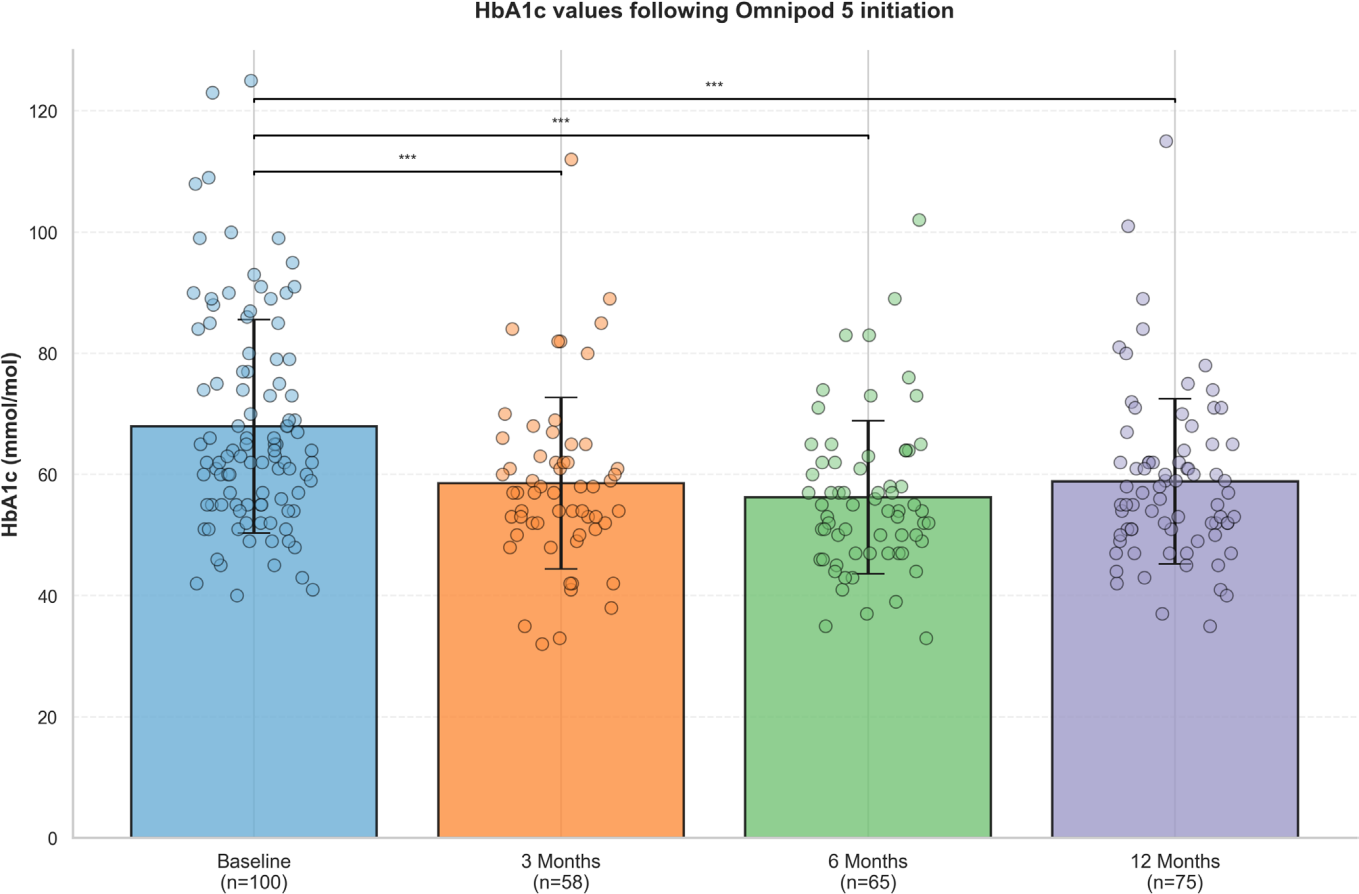
HbA1c values following Omnipod 5 initiation. Data analysed using paired *t*‐tests with data presented as mean and standard deviation. Significant reduction in mean HbA1c (mmol/mol) demonstrated at 3 (*n* = 58), 6 (*n* = 65) and 12 (*n* = 75) months compared to paired baseline data. Two‐tailed paired *p*‐values: < 0.001***.

Significant reductions in mean HbA1c were observed at 3 (*n* = 58), 6 (*n* = 65) and 12 (*n* = 75) months (Figure 2). At 12 months, mean HbA1c improved from 68.5 to 58.8 mmol/mol ([−12.6, −6.7], *p* < 0.001). While reductions in the MDI sub‐group were more pronounced, with HbA1c falling from 71.2 to 57.7 mmol/mol ([−17.3, −9.6], *n* = 41, *p* < 0.001) over the same period, 12‐month HbA1c values were comparable to the overall population (Table S3).

### 3.5 Anthropometric Data

Anthropometric outcomes were initially analysed across all Omnipod 5 users. While there was a significant increase in mean weight at 3 months from 74.4 to 76.4 kg ([1.2, 2.7], *n* = 52, *p* < 0.001), differences did not remain significant at 6 months (75.4 to 75.6 kg, [−2.3, 2.8], *n* = 71, *p* = 0.838) or 12 months (77.0 to 78.3 kg, [−0.2, 2.6], *n* = 70, *p* = 0.093). There was no statistically significant change in paired BMI data across 3‐, 6‐ or 12‐month follow‐up.

Review of medication records indicated that 10 individuals initiated tirzepatide during the 12‐month follow‐up period as adjunctive therapy in T1D. Of these, three had switched from another glucagon‐like peptide‐1 receptor agonist (GLP‐1RA), while the remaining seven were GLP‐1RA naïve. A sensitivity analysis removing these tirzepatide users (*n* = 10 removed) revealed lower mean weights in the non‐tirzepatide sub‐group at all timepoints (Tables S4a and S4b). As observed in the wider cohort, there was a statistically significant increase in weight at 3 months from 71.2 to 73.0 kg ([1.0, 2.7], *n* = 45, *p* < 0.001). While the mean non‐tirzepatide sub‐group weight stabilised at 6 months, a statistically significant increase was noted at 12 months (73.8–75.8, [0.7, 3.4], *n* = 61, *p* = 0.003). Mean BMI remained lower in this non‐tirzepatide sub‐group and did not change significantly at 3 (*n* = 45), 6 (*n* = 62) or 12 months (*n* = 60).

### 3.6 Admissions and Adverse Events

Nine individuals discontinued Omnipod 5 over the 12‐month follow‐up period. Reasons for HCL discontinuation included user preference (*n* = 3), safety concerns related to user error (*n* = 2), connectivity issues (*n* = 1), pregnancy (*n* = 1), skin site reactions (*n* = 1), and moving abroad (*n* = 1). Additional adverse events comprised user error (inappropriate mode or bolusing; *n* = 7), handset dysfunction (*n* = 6), skin site reactions (*n* = 5), difficulties with pod application (*n* = 5), alarm fatigue (*n* = 4), connectivity issues (*n* = 2) and sensor dysfunction (*n* = 1).

Within our cohort, there were three episodes of severe hypoglycaemia requiring third party assistance (0.03 PPPY), but there were no hypoglycaemia‐related admissions. This represented a clinically meaningful reduction compared to the preceding year, where eight episodes of severe hypoglycaemia (0.08 PPPY) and three hypoglycaemia‐related admissions occurred (0.03 PPPY). None of the individuals who initiated Omnipod 5 with concomitant long‐acting insulin reported severe hypoglycaemia.

Six hospital admissions for hyperglycaemia or DKA were recorded (0.06 PPPY). These included four emergency department visits due to hyperglycaemia and two inpatient admissions due to DKA. None of these cases were attributed to pump failure. Overall, this was a reduction from the 15 admissions for hyperglycaemia or DKA in the year before HCL initiation (0.15 PPPY).

### 3.7 Diabetic Eye Disease

Prior to Omnipod 5 start, 67 participants had evidence of diabetic retinopathy, with 30 individuals under the care of specialist ophthalmology services. For the 71 individuals with paired screening data (median follow‐up 325 days, IQR 229–386), retinopathy trajectories were categorised as stable, improved, or progressed relative to baseline. There were no significant differences in baseline demographics between those with follow‐up retinopathy data (completers) to those without (non‐completers) (Table S5).

**FIGURE 3:**
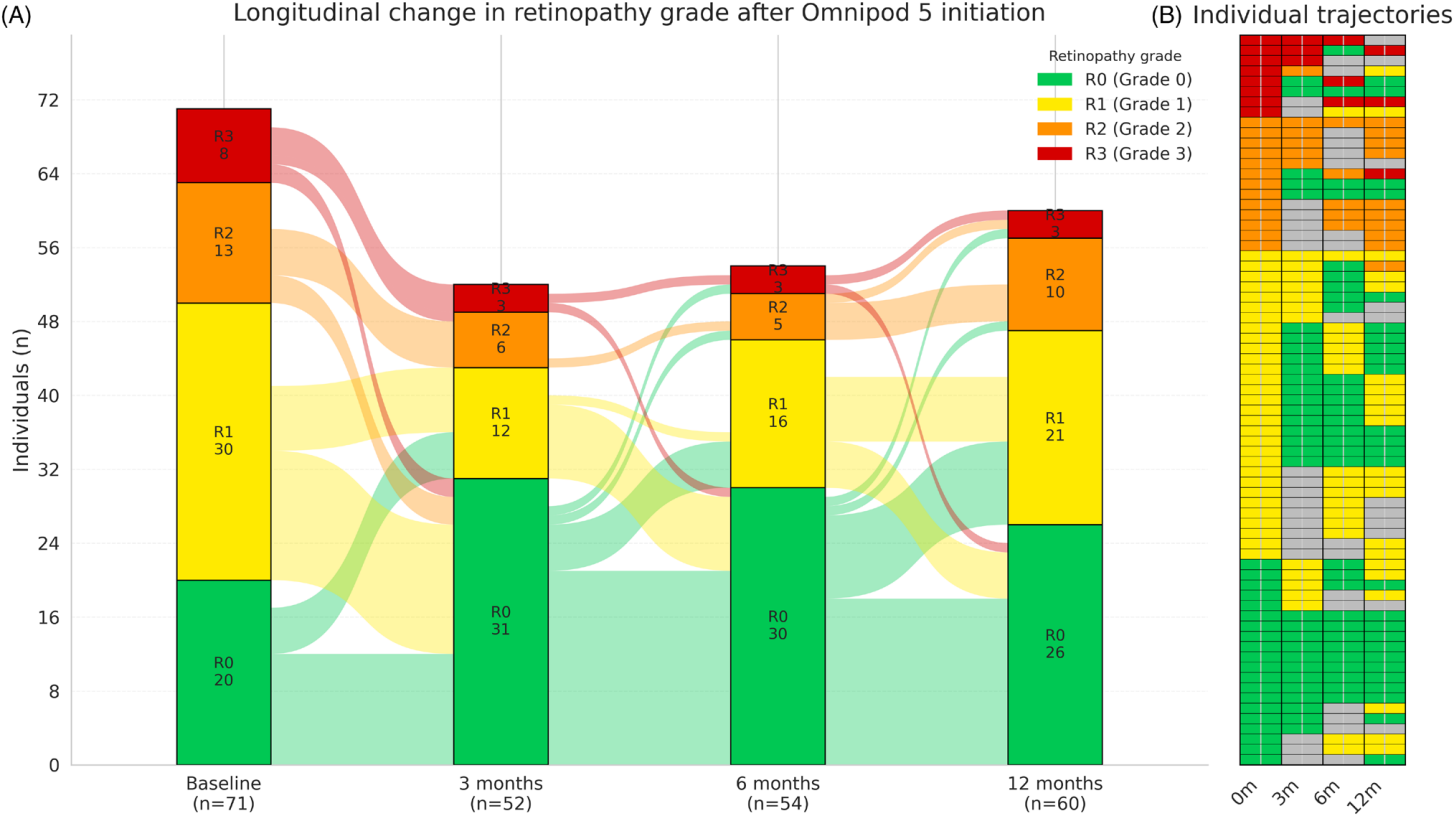
Longitudinal change in retinopathy grade after Omnipod 5 initiation. Retinopathy graded R0–R3: No retinopathy (R0), background retinopathy (R1), pre‐proliferative retinopathy (R2), and proliferative retinopathy (R3). (A) Sankey diagram illustrating longitudinal change. *Y*‐axis represents the number of individuals with paired baseline and follow‐up gradings. Stacked bars show the distribution of retinopathy grades at each timepoint. Connecting bands illustrate longitudinal transitions between grades over time. (B) Individual trajectories depicted in heatmap. The majority remained stable (*n* = 36), with cases of improvement (*n* = 25) and progression (*n* = 10). Notably, none of the participants who discontinued Omnipod 5 did so due to progression of diabetes related eye disease.

Stable retinopathy was observed in the majority of participants (*n* = 36; 51%). Reassuringly, 25 individuals (35%) demonstrated improvement over the 12‐month period (Figure 3).

Progression, defined as any worsening in retinopathy grade relative to baseline, was observed in 10 participants (14%), all of whom exhibited a one‐grade deterioration. Only one individual, with a baseline HbA1c of > 120 mmol/mol, who progressed from R2 to R3A (active proliferative retinopathy), required treatment for proliferative disease. This individual received staged bilateral panretinal photocoagulation 18 months after Omnipod 5 initiation. At the time of writing (February 2026), no further deterioration had been observed in early progressors with recent screening results (*n* = 3).

**FIGURE 4:**
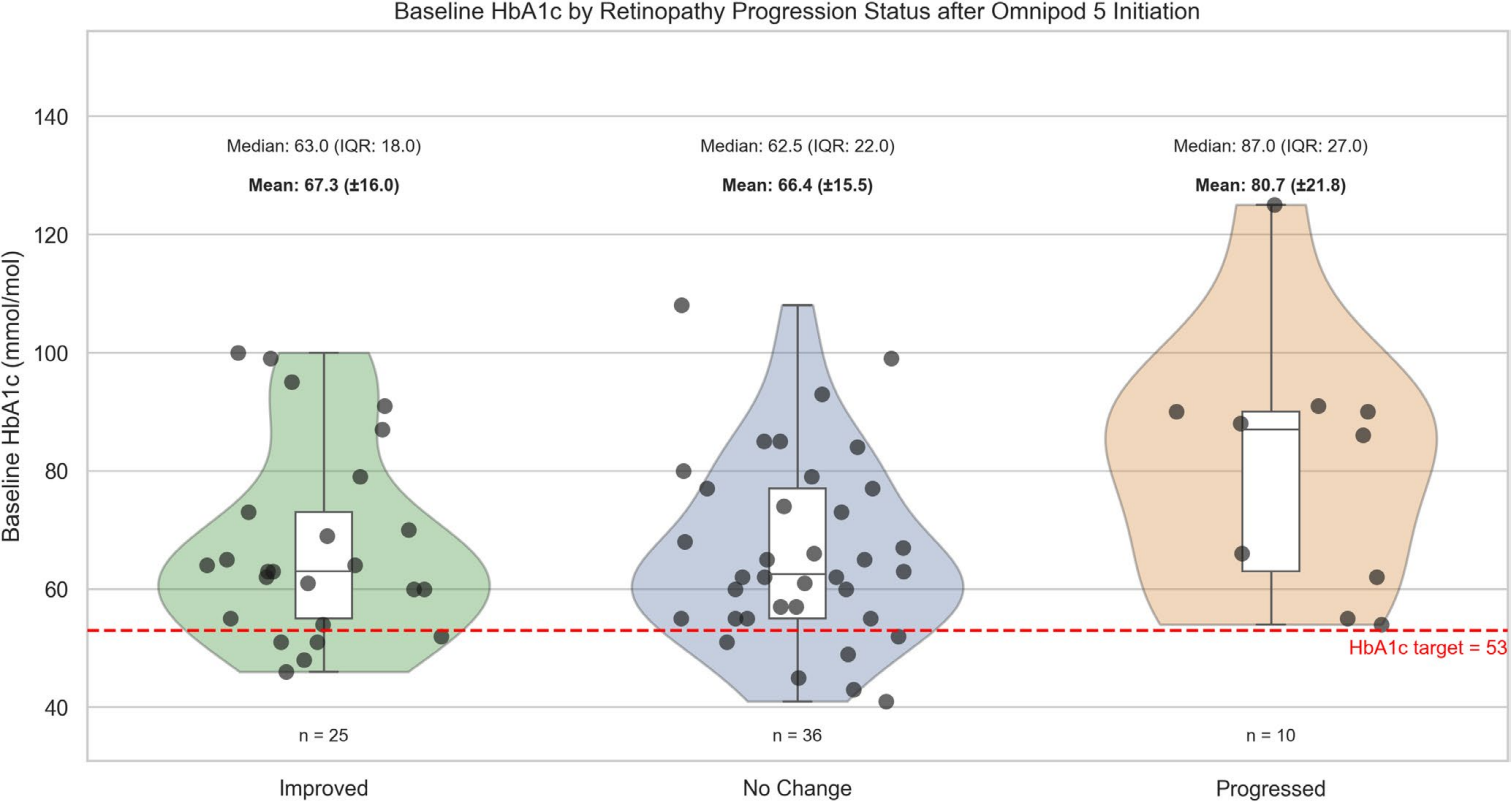
Baseline HbA1c (mmol/mol) by retinopathy progression status after Omnipod 5 initiation. Retinopathy outcomes were categorised into 3 groups: Improved, stable and progressed. Progression was defined as any increase in retinopathy grade relative to baseline, at any follow‐up timepoint. Target HbA1c 53 mmol/mol marked in red. Each data point represents an Omnipod 5 user with both paired retinopathy and baseline HbA1c data (*n* = 71). Mean (SD) and median (IQR) shown. Group differences were analysed using a Kruskal–Wallis test (*H* = 3.70, *p* = 0.157). Notably, no participant with an on‐target baseline HbA1c (< 53 mmol/mol) demonstrated progression of retinopathy.

Exploratory analyses were used to assess correlations between baseline retinopathy, HbA1c, age and retinopathy progression (Figure S2). Baseline age was positively correlated with baseline retinopathy (*r* = 0.47; *p* < 0.001), while a negative correlation was observed between baseline retinopathy and retinopathy progression (*r* = −0.35; *p* = 0.003). The latter correlation is driven by the baseline distribution; specifically, 80% (*n* = 8) of progressors moved from R0 to R1, with 1 from R1 to R2 and 1 from R2 to R3 (Table S6). Preliminary trends also indicated a significant correlation between baseline HbA1c and retinopathy progression (*r* = 0.29, *p* = 0.016). To further explore this relationship, baseline HbA1c results were stratified by retinopathy progression status. No participant with an on‐target baseline HbA1c (< 53 mmol/mol) demonstrated progression of retinopathy in our cohort. However, mean baseline HbA1c did not differ significantly between those who progressed, improved, or remained stable (Figure 4).

### 3.8 Cardiometabolic Parameters

Analysis of paired lipid profiles revealed a statistically significant reduction in triglyceride levels from 0.95 to 0.80 mmol/L (*Z* = −2.04, *p* = 0.041, *n* = 83) over a median follow‐up of 415 days (IQR 314–568). There were no significant changes observed in other components of the lipid profile (Table S7a). Review of medication records confirmed that 29 individuals were prescribed statins at baseline, with no participants commencing a new statin prescription during the follow‐up period. Of those with available dose data (*n* = 21), the majority remained on a stable dose throughout follow‐up (*n* = 16). Five individuals underwent intensification of statin therapy, with a dose increase (*n* = 4) or statin switch (*n* = 1) over the course of follow‐up.

Sensitivity analyses removing (1) tirzepatide users; *n* = 10, (2) individuals with intensification of statin therapy; *n* = 5 and (3) both sub‐groups (*n* = 14 removed) were performed (Tables S7b–S7d). While the reduction in triglycerides remained robust following exclusion of tirzepatide users (*p* = 0.036), this signal was attenuated after excluding those undergoing intensification of statin therapy (*p* = 0.125) and in the combined sub‐group (*p* = 0.105).

Blood pressure remained stable after initiating Omnipod 5. At baseline, median blood pressure was 125/78 mmHg (systolic 117–134, diastolic 73–85, *n* = 64). After a median follow‐up period of 313 days (IQR 249–420, *n* = 57), blood pressure stayed within target at 129/78 mmHg (systolic 117–136, diastolic 72–82), exhibiting no significant change relative to baseline.

### 3.9 Renal Function

Paired urine albumin‐creatinine ratio (uACR) data were available for 78 individuals, obtained at a median follow‐up timepoint of 334.5 (248–41) days. Median uACR remained unchanged from baseline (0.5 mg/mmol, IQR 0.0–1.10) to follow‐up (0.5 mg/mmol, IQR 0.0–1.25). There was no significant change compared to baseline (*z* = −0.383, *p* = 0.702). Of the 78 individuals with paired data, 33 had a decrease in their uACR, 27 an increase, and in 18 cases no change was observed.

Similarly, eGFR remained stable (median 90 mL/min/1.73m^2^, IQR 90–90, *n* = 83), with no significant change (*Z* = 1.88, *p* = 0.235) at a median follow‐up time point of 358.5 (267–450) days.

## 4 Discussion

In this single‐centre retrospective analysis, initiation of the Omnipod 5 HCL system was associated with clinically meaningful benefits in pwT1D. The principal findings were that: (i) HbA1c and CGM‐derived metrics improved significantly by 3 months and were durable to 12 months; (ii) these improvements occurred without any increase in TBR or severe hypoglycaemia; (iii) the greatest glycaemic improvements were observed in those with the lowest baseline TIR; (iv) a small subgroup exhibited progression of diabetic retinopathy, which may or may not represent the phenomenon of early worsening, whilst the retinopathy status improved in a larger proportion and (v) preliminary data demonstrating reduced triglycerides and only transient weight gain were confounded by concomitant medications, highlighting the need for robust prospective evaluation of cardiometabolic risk markers. Importantly, these results were accompanied by a reduction in hospital admissions and acceptable safety outcomes. Taken together, these findings support the real‐world effectiveness, tolerability and safety of Omnipod 5 and offer novel insights into clinical outcomes beyond glycaemia.

This work provided a valuable opportunity to assess glycaemic outcomes in under‐researched populations, including those with high baseline HbA1c or history of frequent severe hypoglycaemia [2, 8]. This cohort had a high proportion transitioning from MDI and a mean baseline HbA1c of 68 (±18.0) mmol/mol, demographics which fall at the upper end of ranges in UK service evaluations and exceed those in pivotal pump trials [2, 26, 27]. The early glycaemic trends observed mirror those reported in other UK cohorts at 10 weeks, 1 month and 3 months, with significant improvements (*p* < 0.001) in HbA1c, TAR, TIR and TBR < 3.0 mmol/L by 3 months [20, 21, 28]. Importantly, improvements in TIR were achieved equitably across the cohort, with no significant correlation observed between ΔTIR and IMD decile. Although this cohort had no socioeconomic stratification by TIR at baseline, these findings suggest socioeconomic deprivation did not dictate glycaemic improvements following Omnipod 5 initiation, echoing the conclusions of other contemporary UK analyses [9, 29].

In contrast to other published UK‐derived evaluations of Omnipod 5, the methodology in this study permitted comparison of paired CGM metrics at 3, 6 and 12 months. This temporal granularity enabled assessment of both the trajectory and durability of glycaemic improvements, rather than relying on a single post‐initiation time point. Paired data revealed that the magnitude of glycaemic improvement seen at 3 months was reflected in the 12‐month data (43.4%–58.3%; Δ + 14.9%), with no further increase over the subsequent 9 months. In keeping with other real‐world cohorts, an inverse association between baseline TIR and ΔTIR at 12 months was observed [27]. This relationship may explain why the magnitude of ΔTIR observed aligns with other real‐world analyses (reporting 13%–18% ΔTIR) and exceeds the 9.3% described in the pivotal HCL trials [2, 9, 20, 27, 28, 30]. Despite these relative benefits, a moderate correlation persisted between baseline TIR and TIR at 12 months (*r* = 0.44, *p* < 0.001), indicating that while individuals with elevated baseline glycaemia experienced the greatest improvements, many still did not achieve international consensus targets for TIR [6]. Similarly, glycaemic variability remained above target (coefficient of variation 35.8%), suggesting broader clinical and sociodemographic factors influence real‐world HCL use. These findings hold relevance for maximising real‐world benefit while minimising the risk of complications such as diabetic retinopathy, where both baseline glycaemia and rapid improvements following HCL initiation may influence risk [31].

Review of eye screening data identified 25 (35%) individuals who exhibited improved retinopathy after Omnipod 5 initiation, with a minority (*n* = 10; 14%) showing progression. Comparing the progression rate from our relatively small cohort to historical data from DCCT and contemporary analyses of other HCL systems remains challenging due to heterogeneous grading systems and variable follow‐up intervals [13, 14, 15, 16, 17, 18]. Reassuringly, all individuals who progressed experienced only a 1‐grade worsening (*n* = 10), with 80% experiencing the least clinically significant change from R0 to R1, and just 1 of the 30 participants, who was already under ophthalmology care prior to initiation, requiring new treatment for proliferative disease. These mild increases, occurring mainly in individuals without baseline retinopathy, mirror the findings of Karakus et al. [14]. Exploratory analyses revealed no individuals with an on‐target (< 53 mmol/mol) baseline HbA1c demonstrated retinopathy progression. While this finding remains inherently exploratory and hypothesis‐generating, it suggests risk may be concentrated among those with off‐target glycaemic metrics at initiation. This is consistent with contemporary data which found that baseline HbA1c rather than ΔHbA1c predicted worsening [14]. Due to the retrospective design, relatively short 12‐month follow‐up, and absence of a control group, it is not possible to determine whether the observed progressions are temporary or permanent, or definitively conclude whether they reflect true EWDR triggered by improved glycaemia, metabolic memory from prior hyperglycaemia, or natural disease progression independent of HCL use [31, 32]. While the ongoing prospective study, INSIGHT 1, will be vital to evaluate whether HCL initiation is associated with EWDR and maculopathy, as well as exploring mechanisms behind this phenomena, longer‐term analyses are required to address the metabolic memory hypothesis [33]. Clinically, these data highlight the need to identify high‐risk individuals and determine whether structured onboarding, with additional retinal screening and optimised HCL settings should be established for these individuals.

In addition to incorporating data on diabetic eye disease, future studies should also examine the cardiometabolic effects of Omnipod 5, including changes in blood pressure, lipid profiles and urinary ACR, an important predictor of long‐term cardiometabolic risk [34, 35]. While this study was not prospectively powered to detect cardiometabolic changes and included a relatively low‐risk cohort with on‐target baseline profiles, these preliminary data do align with previous research. Notably, the observed pattern of an isolated triglyceride improvement is consistent with data from the DCCT cohort, where intensive insulin therapy resulted in significant triglyceride reductions without any significant changes in low‐density lipoprotein cholesterol or high‐density lipoprotein cholesterol [36]. Mechanistically, these triglyceride reductions likely reflect favourable shifts in insulin delivery profiles following Omnipod 5 initiation, where changes in insulin availability could result in suppression of hepatic very low‐density lipoprotein production and increased lipoprotein lipase‐dependent clearance of triglycerides [37].

However, it is important to interpret these findings in the context of the modest absolute triglyceride change (median 0.95–0.80 mmol/L; *p* = 0.041) and the sensitivity analyses performed. While the reduction in triglycerides remained significant following exclusion of GLP‐1RA users (*n* = 10), sensitivity analyses removing individuals undergoing statin intensification (*n* = 5 removed) attenuated this signal (*p* = 0.125). In practice, it is difficult to determine whether the statistically significant reduction in triglycerides observed in the overall cohort was attributable to the metabolic effects of Omnipod 5 or driven primarily by concomitant statin therapy. Similarly, the weight changes observed should be interpreted in the context of the tirzepatide sensitivity analysis, which indicated that tirzepatide masked modest (mean + 2.0 kg) but statistically significant weight gain in non‐tirzepatide users at 12‐months. The significant weight gain observed in these individuals was likely attributable to the rapidly improved glycaemia, a well‐documented phenomenon following intensification of insulin therapy [38]. Collectively, these data highlight the need for larger, prospectively powered studies to isolate the cardiometabolic impacts of HCL from the effects of concomitant therapies, across cohorts with varying baseline cardiometabolic risk. Given that cardiovascular disease remains the leading cause of morbidity and mortality for pwT1D, this represents a vital focus for the field [19].

### 4.1 Strengths and Limitations

In the context of the findings discussed above, the following strengths and weaknesses merit consideration. First, the current literature on HCL systems is overwhelmingly focused on glycaemic outcomes. Uniquely, this study provides a novel perspective by integrating routinely collected data on lipid profiles, renal function and diabetic eye disease. This addresses a key evidence gap and facilitates exploration of whether HCL use is associated with changes in cardio‐renal‐microvascular risk markers beyond glycaemic control. Notably, the sample was highly representative of the wider clinic population. The sociodemographic characteristics of this cohort mirror those reported by Anandhakrishnan et al., with an identical proportion of participants in the two most deprived IMD quintiles (48%) and comparable ethnicity data [9]. These factors maximise the generalisability of the results to the wider UK population.

Key limitations include the retrospective design, modest sample size, and lack of control group, which collectively limit causal inference and external validity. Sensitivity analyses also demonstrated that concomitant medications influenced the trends observed. Additionally, with missing paired data, the role of attrition bias should be considered for key outcomes. While missing CGM data was minimal, gaps may correspond to lower HCL adherence and wear‐time, potentially overestimating true glycaemic improvements. Conversely, in the retinopathy analysis, individuals without retinopathy were likely to be on longer screening recall intervals and thus have missing data, potentially underestimating the true progression rate. Finally, while the paired CGM data were granular, data on individualised HCL system settings were not collected. Nevertheless, these findings extend the current evidence base on HCL systems beyond glycaemic outcomes but should ultimately be considered preliminary and warrant further prospective evaluation.

## 5 Conclusions

Real‐world use of Omnipod 5 is associated with significant and sustained improvements in glycaemic control at 12 months. Notably, this study is the first to report outcomes on diabetic eye disease following Omnipod 5 initiation. Preliminary data suggest retinopathy progression is confined to a minority of individuals who may initiate Omnipod 5 with off‐target baseline glycaemic metrics. Further prospective, multi‐centre studies are required for comparison with non‐HCL cohorts and to determine whether the observed retinal changes are transient or represent EWDR, metabolic memory, or natural disease progression. Finally, future longitudinal studies of cardiometabolic risk markers should account for concomitant real‐world therapies to evaluate and optimise real‐world outcomes beyond glycaemic metrics.

## Funding

The authors have nothing to report.

## Conflicts of Interest

S.B. has received research support from Tandem Diabetes Care Inc. (San Diego, CA) and Dexcom Inc. (San Diego, CA). Has received travel grants through charities sponsored by Eli Lilly and Company (Indianapolis, IN) and Abbott UK (Maidenhead, UK). J.E. has received investigator‐led funding from Dexcom and has received honoraria from Abbott, Boehringer Ingelheim, Dexcom, Eli Lilly, Glooko, Insulet, Medtronic, Novo Nordisk, Roche, Sanofi, and Ypsomed as advisory board or speaker fees. A.I. has received investigator‐led funding from Dexcom, Abbott, and has received honoraria from Eli Lilly, Sanofi, Boehringer Ingelheim, Ypsomed, and Diiachi Sankyo as speaker fees. E.M., I.G., R.M., J.M., D.S., and H.W.T. declare no conflicts of interest.

## Supporting information


**Figure S1:** Methodology and summary of 12‐month sample sizes. One hundred Omnipod 5 users were randomly selected from the wider clinic population (*N* = 377). Paired data varied across outcomes and timepoints due to the nature of the routine clinical dataset. Sensitivity analyses were performed to address missing data and use of concomitant medications. Abbreviations: type 1 diabetes (T1D), continuous glucose monitoring (CGM), body mass index (BMI) and Glucagon‐Like Peptide‐1 Receptor Agonists (GLP‐1RA).
**Table S1:** Ethnicity data for Omnipod 5 starters (*N* = 100). Ethnicity groups presented in line with the Office for National Statistics 2021 Census results.
**Table S2:** Paired changes in glycaemic metrics following Omnipod 5 initiation. Paired *t*‐tests used to compare baseline with follow‐up results. Individuals who discontinued Omnipod 5 were excluded from the analysis. Abbreviations: standard deviation (SD), time above range (TAR), time in range (TIR), time below range (TBR), coefficient of variation (CV), glucose management indicator (GMI), confidence interval (CI). *p*‐values are two‐sided (*p < 0.05, **p < 0.01, ***p < 0.001).
**Table S3:** Paired HbA1c (mmol/mol) data for pump naïve subgroup transitioning from multiple daily injections (MDI). Data analysed using paired *t*‐tests and presented as mean with 95% confidence interval. Two‐tailed *p*‐values are shown (*p < 0.05, **p < 0.01, ***p < 0.001).
**Table S4a:** Anthropometric data across full cohort who continued Omnipod 5 for duration of 12‐month follow‐up period. Sample included *n* = 10 who were prescribed tirzepatide.
**Table S4b:** Anthropometric data across non‐tirzepatide subgroup (*n* = 10 tirzepatide users removed). Individuals established on tirzepatide prior to Omnipod 5 initiation, or who started a new tirzepatide prescription at any point during the 12‐month follow‐up were removed.
**Table S5:** Baseline demographics and clinical characteristics of completers and non‐completers for retinopathy outcomes. Completers = individuals who had paired baseline and follow‐up retinopathy gradings. Non‐completers = individuals without paired data, with absent baseline or follow‐up retinopathy gradings. Retinopathy status was assessed as part of the NHS Diabetes Eye Screening Programme and graded R0–R3. Continuous variables presented as median (IQR). Categorical variables presented as *n* (%). Comparisons between groups were performed using Mann–Whitney *U* test or chi‐square test.
**Figure S2:** Correlation matrix illustrating potential risk factors for progression of diabetic retinopathy. Heatmap depicts Pearson correlation coefficients between baseline diabetic retinopathy grade (R0–R3), baseline HbA1c (mmol/mol), baseline age and retinopathy progression (binary yes = 1, no = 0). Paired retinopathy gradings available for *n* = 71. Individual cells correspond to Pearson correlation coefficients. Statistical significance noted as two‐sided *p*‐values *p < 0.05, **p < 0.01, ***p < 0.001. Baseline age was positively correlated with baseline retinopathy (*r* = 0.47; *p* < 0.001). A negative correlation was observed between baseline retinopathy and retinopathy progression (*r* = −0.35; *p* = 0.003). Data also indicated a correlation between baseline HbA1c and retinopathy progression (*r* = 0.29, *p* = 0.016).
**Table S6:** Retinopathy outcomes stratified by baseline retinopathy grades. Retinopathy status was assessed as part of the NHS Diabetes Eye Screening Programme and graded R0‐R3. Baseline glycaemic data for HbA1c (mmol/mol) and time in range (TIR) between 3.9 and 10.0 mmol/L (%) also shown. Change from baseline calculated for paired data points at baseline and 12 months. Normally distributed variables summarised as mean ± standard deviation (SD). Only participants with baseline retinopathy gradings and at least one follow‐up assessment at 3, 6 or 12 months were included. Retinopathy progression was defined as any follow‐up grading greater than baseline grading at any follow‐up time point. Improvement was defined as any follow‐up grading less than baseline in the absence of progression.
**Table S7a:** Changes in lipid profiles relative to baseline after commencing Omnipod 5. At 12 months the most recent follow‐up lipid profile was compared to baseline (median follow‐up 415 days; IQR 314–568). Wilcoxon signed‐rank tests were used to compare paired values. Two‐sided *p*‐values *p < 0.05. HDL = high‐density lipoprotein.
**Table S7b:** Changes in lipid profiles relative to baseline after commencing Omnipod 5 with tirzepatide users (*n* = 10) removed. At 12 months the most recent follow‐up lipid profile was compared to baseline. Wilcoxon signed‐rank tests were used to compare paired values. Two‐sided *p*‐value *p < 0.05. HDL = high‐density lipoprotein.
**Table S7c:** Changes in lipid profiles relative to baseline after commencing Omnipod 5 with individuals undergoing intensification of statin therapy over the course of follow‐up removed (*n* = 5). At 12 months the most recent follow‐up lipid profile was compared to baseline. Wilcoxon signed‐rank tests were used to compare paired values. Two‐sided *p*‐value *p < 0.05. HDL = high‐density lipoprotein.
**Table S7d:** Changes in lipid profiles relative to baseline after commencing Omnipod 5 with individuals on tirzepatide or undergoing either intensification of statin therapy over the course of follow‐up removed (combined *n* = 14 removed). At 12 months the most recent follow‐up lipid profile was compared to baseline. Wilcoxon signed‐rank tests were used to compare paired values. Two‐sided *p*‐value *p < 0.05. HDL = high‐density lipoprotein.

## Data Availability

The data that were used in this study are not publicly available as they were collected as part of routine clinical care. Anonymised data are available from the corresponding author upon reasonable request.
