# Supplementary material for: Looking Beyond Glycaemic Control: Real‐World 12‐Month Cardiometabolic and Retinopathy Outcomes Following Omnipod 5 Initiation in People With Type 1 Diabetes
Source: Diabetes Obes Metab. 2026 Jul 20;28(10):9166–77. doi: 10.1111/dom.71129 (PMC13538799; doi:10.1111/dom.71129)
Supplement: Supplementary file 1 [file DOM-28-9166-s001.docx]

**Supplementary Appendix**

**Supplementary Figure 1.** Methodology and summary of 12-month sample sizes. One hundred Omnipod 5 users were randomly selected from the wider clinic population (N=377). Paired data varied across outcomes and timepoints due to the nature of the routine clinical dataset. Sensitivity analyses were performed to address missing data and use of concomitant medications. Abbreviations: type 1 diabetes (T1D), continuous glucose monitoring (CGM), body mass index (BMI) and Glucagon-Like Peptide-1 Receptor Agonists (GLP-1RA).

| **Ethnicity** | **Count (n=)** |
| --- | --- |
| **White:** | |
| English, Welsh, Scottish, Northern Irish or British | 73 |
| Any other White background | 3 |
| **Asian or Asian British:** | |
| Pakistani | 3 |
| Chinese | 1 |
| **Black, Black British, Caribbean or African:** | |
| African | 1 |
| Caribbean | 1 |
| Any other Black, Black British, or Caribbean background | 2 |
| **Mixed or multiple ethnic groups:** | |
| White and Black African | 2 |
| **Other ethnic group:** | |
| Any other ethnic group | 6 |
| **Unknown:** | |
| Not stated/unknown | 8 |

**Supplementary Table 1.** Ethnicity data for Omnipod 5 starters (N=100). Ethnicity groups presented in line with the Office for National Statistics 2021 Census results.

| Glycaemic metric | Timepoint  (months) | Baseline mean | Follow-up mean | Mean difference [95% CI] | Two-sided p value | Paired samples (n=) |
| --- | --- | --- | --- | --- | --- | --- |
| **TAR >13.9 mmol/L (%)** | 3 | 27.6 | 16.1 | -11.5 [-15.0, -8.1] | <0.001*** | 88 |
|  | 6 | 27.7 | 16.9 | -10.8 [-15.1, -6.5] | <0.001*** | 87 |
|  | 12 | 28.2 | 17.3 | -10.9 [-15.5, -6.2] | <0.001*** | 86 |
| **TAR 10.1–13.9 mmol/L (%)** | 3 | 26.3 | 22.5 | -3.8 [-6.0, -1.7] | <0.001*** | 88 |
|  | 6 | 26.3 | 23.3 | -3.0 [-5.6, -0.4] | =0.023* | 87 |
|  | 12 | 26.3 | 22.8 | -3.5 [-5.9, -1.2] | =0.004** | 86 |
| **TIR 3.9–10.0 mmol/L (%)** | 3 | 44.0 | 59.7 | 15.7 [11.9, 19.5] | <0.001*** | 88 |
|  | 6 | 43.9 | 58.3 | 14.4 [10.2, 18.6] | <0.001*** | 87 |
|  | 12 | 43.4 | 58.3 | 14.9 [10.6, 19.2] | <0.001*** | 86 |
| **TBR 3.0–3.8 mmol/L (%)** | 3 | 1.5 | 1.3 | -0.2 [-0.6, 0.2] | =0.246 | 88 |
|  | 6 | 1.5 | 1.2 | -0.3 [-0.7, 0.0] | =0.054 | 87 |
|  | 12 | 1.5 | 1.2 | -0.3 [-0.6, 0.0] | =0.082 | 86 |
| **TBR <3.0 mmol/L (%)** | 3 | 0.7 | 0.4 | -0.3 [-0.5, -0.1] | =0.006** | 88 |
|  | 6 | 0.7 | 0.3 | -0.4 [-0.6, -0.2] | <0.001*** | 87 |
|  | 12 | 0.7 | 0.3 | -0.4 [-0.6, -0.2] | <0.001*** | 86 |
| **Mean glucose (mmol/L)** | 3 | 11.4 | 9.9 | -1.5 [-1.9, -1.0] | <0.001*** | 79 |
|  | 6 | 11.4 | 10.1 | -1.3 [-1.9, -0.8] | <0.001*** | 78 |
|  | 12 | 11.5 | 10.0 | -1.5 [-2.1, -0.9] | <0.001*** | 77 |
| **SD (mmol/L)** | 3 | 4.0 | 3.7 | -0.3 [-0.6, -0.1] | =0.005** | 72 |
|  | 6 | 4.0 | 3.6 | -0.4 [-0.6, -0.1] | =0.003** | 70 |
|  | 12 | 4.0 | 3.6 | -0.4 [-0.7, -0.2] | <0.001*** | 70 |
| **CV (%)** | 3 | 35.8 | 36.3 | 0.5 [-0.7, 1.8] | =0.414 | 87 |
|  | 6 | 35.7 | 35.7 | 0.0 [-1.4, 1.3] | =0.950 | 86 |
|  | 12 | 35.8 | 35.8 | 0.0 [-1.4, 1.4] | =0.965 | 85 |
| **GMI (mmol/mol)** | 3 | 63.8 | 57.0 | -6.8 [-9.4, -4.0] | <0.001*** | 57 |
|  | 6 | 63.4 | 56.3 | -7.1 [-9.4, -4.9] | <0.001*** | 48 |
|  | 12 | 64.6 | 57.2 | -7.4 [-9.1, -5.7] | <0.001*** | 56 |
| HbA1c (mmol/mol) | 3 | 67.7 | 58.5 | -9.2 [-11.6, -6.7] | <0.001*** | 58 |
|  | 6 | 67.6 | 56.2 | -11.4 [-13.8, -8.9] | <0.001*** | 65 |
|  | 12 | 68.5 | 58.8 | -9.7 [-12.6, -6.7] | <0.001*** | 75 |

**Supplementary Table 2.** Paired changes in glycaemic metrics following Omnipod 5 initiation. Paired t-tests used to compare baseline with follow-up results. Individuals who discontinued Omnipod 5 were excluded from the analysis. Abbreviations: standard deviation (SD), time above range (TAR), time in range (TIR), time below range (TBR), coefficient of variation (CV), glucose management indicator (GMI), confidence interval (CI). p-values are two-sided (*p<0.05, **p<0.01, ***p<0.001).

| **Timepoint** | **Mean HbA1c (mmol/mol) at baseline** | **Mean HbA1c (mmol/mol) at follow up** | **Mean difference (mmol/mol) [95% CI]** | **Two-sided p value** | **Paired samples**  **(n=)** |
| --- | --- | --- | --- | --- | --- |
| 3 months (+/- 6 weeks) | 68.7 | 60.1 | -8.6  [-11.5, -5.6] | <0.001*** | 31 |
| 6 months (+/- 12 weeks) | 69.1 | 57.6 | -11.5  [-15.0, -8.1] | <0.001*** | 38 |
| 12 months (+/-12 weeks) | 71.2 | 57.7 | -13.5  [-17.3, -9.6] | <0.001*** | 41 |

**Supplementary Table 3.** Paired HbA1c (mmol/mol) data for pump naïve subgroup transitioning from multiple daily injections (MDI). Data analysed using paired t-tests and presented as mean with 95% confidence interval. Two-tailed p-values are shown (*p<0.05, **p<0.01, ***p<0.001).

| Outcome | Follow-up (months) | n (paired) | Baseline mean | Follow-up mean | Mean change (95% CI) | p value |
| --- | --- | --- | --- | --- | --- | --- |
| Weight (kg) | 3 | 52 | 74.4 | 76.4 | 2.0 (1.2, 2.7) | <0.001*** |
|  | 6 | 71 | 75.4 | 75.6 | 0.2 (-2.3, 2.8) | 0.838 |
|  | 12 | 70 | 77.0 | 78.3 | 1.3 (-0.2, 2.6) | 0.093 |
| BMI (kg/m²) | 3 | 52 | 26.8 | 27.2 | 0.4 (0.0, 0.9) | 0.053 |
|  | 6 | 71 | 27.4 | 27.0 | -0.4 (-1.6, 0.6) | 0.397 |
|  | 12 | 69 | 27.7 | 27.4 | -0.3 (-1.2, 0.7) | 0.558 |

**Supplementary Table 4a**. Anthropometric data across full cohort who continued Omnipod 5 for duration of 12-month follow-up period. Sample included n=10 who were prescribed tirzepatide.

| Outcome | Follow-up (months) | n (paired) | Baseline mean | Follow-up mean | Mean change (95% CI) | p value |
| --- | --- | --- | --- | --- | --- | --- |
| Weight (kg) | 3 | 45 | 71.2 | 73.0 | 1.8 (1.0, 2.7) | <0.001*** |
|  | 6 | 62 | 72.2 | 73.0 | 0.8 (-1.5, 3.0) | 0.490 |
|  | 12 | 61 | 73.8 | 75.8 | 2.0 (0.7, 3.4) | 0.003* |
| BMI (kg/m²) | 3 | 45 | 25.1 | 25.4 | 0.3 (-0.1, 0.9) | 0.125 |
|  | 6 | 62 | 26.0 | 25.6 | -0.4 (-1.6, 0.7) | 0.453 |
|  | 12 | 60 | 26.1 | 26.0 | -0.1 (-1.2, 1.0) | 0.852 |

**Supplementary Table 4b.** Anthropometric data across non-tirzepatide subgroup (n=10 tirzepatide users removed). Individuals established on tirzepatide prior to Omnipod 5 initiation, or who started a new tirzepatide prescription at any point during the 12-month follow-up were removed.

| Characteristic | Completers  (n=71) | Non-completers  (n=20) | p value |
| --- | --- | --- | --- |
| Age, years | 28.0 (24.0-36.5) | 25 (21.8-38.5) | 0.342 |
| Female sex, n (%) | 44 (62) | 15 (75) | 0.281 |
| IMD, ranks 1-10 | 5.0 (3.0-8.0) | 5.5 (2.0-8.2) | 0.992 |
| BMI, kg/m^2^ | 25.1 (22.2-29.9) | 27.1 (23.3-29.2) | 0.446 |
| Weight, kg | 73.8 (62.4-87.7) | 71.8 (63.4-87.2) | 0.684 |
| Diabetes duration, years | 16.9 (10.2-24.6) | 12.3 (9.3-30.8) | 0.608 |
| HbA1c, mmol/mol | 64.0 (55.0-79.5) | 60.0 (51.2-66.5) | 0.066 |
| Baseline retinopathy grade  R0  R1  R2  R3 | 20 (28)  30 (42)  13 (18)  8 (11) | 7 (35)  7 (35)  1 (5)  2 (10) |  |

**Supplementary Table 5.** Baseline demographics and clinical characteristics of completers and non-completers for retinopathy outcomes. Completers=individuals who had paired baseline and follow-up retinopathy gradings. Non-completers=individuals without paired data, with absent baseline or follow-up retinopathy gradings. Retinopathy status was assessed as part of the NHS Diabetes Eye Screening Programme and graded R0 to R3. Continuous variables presented as median (IQR). Categorical variables presented as n (%). Comparisons between groups were performed using Mann-Whitney U Test or Chi-square test.

**Supplementary Figure 2.** Correlation matrix illustrating potential risk factors for progression of diabetic retinopathy**.** Heatmap depicts Pearson correlation coefficients between baseline diabetic retinopathy grade (R0-R3), baseline HbA1c (mmol/mol), baseline age and retinopathy progression (binary yes=1, no=0). Paired retinopathy gradings available for n=71. Individual cells correspond to Pearson correlation coefficients. Statistical significance noted as two-sided p-values *p<0.05, **p<0.01 and ***p<0.001. Baseline age was positively correlated with baseline retinopathy (r=0.47; p<0.001). A negative correlation was observed between baseline retinopathy and retinopathy progression (r=-0.35; p=0.003). Data also indicated a correlation between baseline HbA1c and retinopathy progression (r=0.29, p=0.016).

| Baseline retinopathy grade | n | Baseline HbA1c mean (SD) | Baseline TIR mean (SD) | ΔHbA1c mean (SD) | ΔTIR mean (SD) | Progressed n (%) | Improved n (%) | Stable n (%) |
| --- | --- | --- | --- | --- | --- | --- | --- | --- |
| R0 | 20 | 66.0 (13.9) | 44.8 (20.7) | -10.8 (10.4) | 21.7 (14.2) | 8 (40.0) | 0 (0.0) | 12 (60.0) |
| R1 | 30 | 69.5 (21.5) | 42.0 (21.4) | -10.7 (12.2) | 17.6 (16.7) | 1 (3.3) | 18 (60.0) | 11 (36.7) |
| R2 | 13 | 66.5 (17.7) | 50.8 (20.8) | -12.1 (14.3) | -0.6 (22.3) | 1 (7.7) | 2 (15.4) | 10 (76.9) |
| R3 | 8 | 74.1 (23.7) | 33.6 (10.8) | -8.1 (9.1) | 20.1 (11.0) | 0 (0.0) | 5 (62.5) | 3 (37.5) |
| Total | 71 | 68.5 (19.0) | 43.5 (20.3) | -10.7 (11.5) | 15.7 (18.1) | 10 (14.1) | 25 (35.2) | 36 (50.7) |

**Supplementary Table 6.** Retinopathy outcomes stratified by baseline retinopathy grades. Retinopathy status was assessed as part of the NHS Diabetes Eye Screening Programme and graded R0-R3. Baseline glycaemic data for HbA1c (mmol/mol) and time in range (TIR) between 3.9–10.0 mmol/L (%) also shown. Change from baseline calculated for paired data points at baseline and 12 months. Normally distributed variables summarised as mean ± standard deviation (SD). Only participants with baseline retinopathy gradings and at least one follow-up assessment at 3, 6, or 12 months were included. Retinopathy progression was defined as any follow-up grading greater than baseline grading at any follow-up time point. Improvement was defined as any follow-up grading less than baseline in the absence of progression.

|  | **Baseline median (IQR)** | **Follow-up median (IQR)** | **Z (Wilcoxon signed rank)** | **p-value** | **Paired values**  **(n=)** |
| --- | --- | --- | --- | --- | --- |
| **Total cholesterol**  **(mmol/L)** | 4.20 (3.70-4.93) | 4.25 (3.63-4.98) | -1.135 | 0.257 | 83 |
| **Triglycerides (mmol/L)** | 0.95 (0.70-1.20) | 0.80 (0.63-1.10) | -2.042 | 0.041* | 83 |
| **HDL cholesterol (mmol/L)** | 1.60 (1.32-1.96) | 1.65 (1.37-1.92) | 0.061 | 0.951 | 83 |
| **Non-HDL cholesterol (mmol/L)** | 2.60 (2.03-3.20) | 2.50 (2.10-3.10) | -1.147 | 0.251 | 80 |
| **Total/HDL cholesterol ratio** | 2.60 (2.20-3.10) | 2.50 (2.13-3.00) | -0.509 | 0.610 | 82 |

**Supplementary Table 7a.** Changes in lipid profiles relative to baseline after commencing Omnipod 5. At 12 months the most recent follow-up lipid profile was compared to baseline (median follow-up 415 days; IQR 314-568). Wilcoxon signed-rank tests were used to compare paired values. Two-sided p-values *p<0.05. HDL = high-density lipoprotein.

|  | **Baseline median (IQR)** | **Follow-up median (IQR)** | **Z (Wilcoxon signed rank)** | **p-value** | **Paired values**  **(n=)** |
| --- | --- | --- | --- | --- | --- |
| **Total cholesterol**  **(mmol/L)** | 4.25 (3.70-4.98) | 4.25 (3.60-4.93) | -0.910 | 0.363 | 73 |
| **Triglycerides (mmol/L)** | 1.00 (0.70-1.20) | 0.80 (0.60-1.10) | -2.095 | 0.036* | 73 |
| **HDL cholesterol (mmol/L)** | 1.59 (2.10-3.20) | 1.63 (1.36-1.88) | 0.101 | 0.920 | 73 |
| **Non-HDL cholesterol (mmol/L)** | 2.60 (2.10 – 3.20) | 2.60 (2.10-3.13) | -0.823 | 0.411 | 72 |
| **Total/HDL cholesterol ratio** | 2.60 (2.20-3.10) | 2.60 (2.30-3.03) | -0.254 | 0.800 | 72 |

**Supplementary Table 7b.** Changes in lipid profiles relative to baseline after commencing Omnipod 5 with tirzepatide users (n=10) removed. At 12 months the most recent follow-up lipid profile was compared to baseline. Wilcoxon signed-rank tests were used to compare paired values. Two-sided p-value *p<0.05. HDL = high-density lipoprotein.

|  | **Baseline median (IQR)** | **Follow-up median (IQR)** | **Z (Wilcoxon signed rank)** | **p-value** | **Paired values**  **(n=)** |
| --- | --- | --- | --- | --- | --- |
| **Total cholesterol**  **(mmol/L)** | 4.20 (3.70-4.90) | 4.20 (3.60-4.90) | -1.068 | 0.286 | 78 |
| **Triglycerides (mmol/L)** | 0.90 (0.65-1.20) | 0.80 (0.60-1.10) | -1.533 | 0.125 | 78 |
| **HDL cholesterol (mmol/L)** | 1.60 (1.34-1.97) | 1.65 (1.36-1.92) | -0.414 | 0.679 | 78 |
| **Non-HDL cholesterol (mmol/L)** | 2.55 (2.00-3.20) | 2.50 (2.08-3.13) | -0.827 | 0.408 | 76 |
| **Total/HDL cholesterol ratio** | 2.55 (2.20-3.08) | 2.50 (2.20-3.00) | -0.028 | 0.978 | 77 |

**Supplementary Table 7c.** Changes in lipid profiles relative to baseline after commencing Omnipod 5 with individuals undergoing intensification of statin therapy over the course of follow-up removed (n=5). At 12 months the most recent follow-up lipid profile was compared to baseline. Wilcoxon signed-rank tests were used to compare paired values. Two-sided p-value *p<0.05. HDL = high-density lipoprotein.

|  | **Baseline median (IQR)** | **Follow-up median (IQR)** | **Z (Wilcoxon signed rank)** | **p-value** | **Paired values**  **(n=)** |
| --- | --- | --- | --- | --- | --- |
| **Total cholesterol**  **(mmol/L)** | 4.20 (3.70-4.90) | 4.25 (3.60-4.90) | -0.664 | 0.506 | 69 |
| **Triglycerides (mmol/L)** | 0.90 (0.70-1.20) | 0.80 (0.60-1.10) | -1.619 | 0.105 | 69 |
| **HDL cholesterol (mmol/L)** | 1.60 (1.31-1.95) | 1.63 (1.36-1.88) | -0.196 | 0.845 | 69 |
| **Non-HDL cholesterol (mmol/L)** | 2.60 (2.00-3.20) | 2.60 (2.10-3.13) | -0.471 | 0.638 | 68 |
| **Total/HDL cholesterol ratio** | 2.60 (2.20-3.10) | 2.60 (2.30-3.03) | 0.175 | 0.861 | 68 |

**Supplementary Table 7d.** Changes in lipid profiles relative to baseline after commencing Omnipod 5 with individuals on tirzepatide or undergoing either intensification of statin therapy over the course of follow-up removed (combined n=14 removed). At 12 months the most recent follow-up lipid profile was compared to baseline. Wilcoxon signed-rank tests were used to compare paired values. Two-sided p-value *p<0.05. HDL = high-density lipoprotein.
